# Association between hyperpyrexia and poststroke outcomes in patients with recanalization after mechanical thrombectomy: a retrospective cohort study

**DOI:** 10.1186/s12883-021-02400-8

**Published:** 2021-09-21

**Authors:** Man Chen, Jinghuan Fang, Xintong Wu, Qin Liu, Ling Feng, Li He

**Affiliations:** 1 Department of Neurology, West China Hospital, Sichuan University, 610041 Chengdu, China; 2 Department of Neurology, West China Hospital, Sichuan University/West China School of Nursing, Sichuan University, 610041 Chengdu, China

**Keywords:** Hyperpyrexia, Mechanical thrombectomy, Recanalization, Acute ischemic stroke

## Abstract

**Background:**

Limited data are available for evaluating the relationship between the prognosis and body temperature (BT) in patients treated with mechanical thrombectomy (MT), especially in those with successful recanalization. We aimed to explore the prognostic value of BT in predicting outcomes of stroke recovery at 3 months poststroke.

**Methods:**

We retrospectively analyzed the relationship among BT levels as a continuous variable, with fever (BT ≥ 37.5℃) as a binary variable, and obtained several outcomes of interest. Subjects were stratified according to successful recanalization (thrombolysis in cerebral infarction scores of 2b-3) following MT. Functional independence was defined as a modified Rankin scale (mRS) score of 0–2.

**Results:**

In total, 258 patients were included. The proportion of patients with functional independence was significantly lower among patients with BT ≥ 37.5℃ than among those with BT < 37.5 °C (45.3 % versus 23.0 %; *P* < 0.001). In the multivariate analysis, hyperpyrexia (especially BT ≥ 38 °C) was significantly associated with poor 3-month outcomes in patients treated with MT. Subgroup analysis was conducted by comparing the successful recanalization group with the non-recanalization group, showing that BT ≥ 37.5 °C was associated with a significantly lower proportion of functional independence in the recanalized patients. Besides, the Kaplan-Meier model showed that the fever group had significantly lower survival rates than the non-fever group during the 3-month follow-up.

**Conclusions:**

In patients treated with MT, hyperpyrexia is an independent predictor of poststroke outcomes at 3 months, particularly in those with successful recanalization.

**Supplementary Information:**

The online version contains supplementary material available at 10.1186/s12883-021-02400-8.

## Background

Stroke remains a significant cause of morbidity and mortality throughout the world, and acute ischemic stroke (AIS) makes up the vast majority of stroke cases [1]. Early recanalization is the main target of treatment for AIS and is associated with good clinical outcomes at 3 months [2]. Recent randomized controlled clinical trials (RCTs) have provided overwhelming evidence of the efficacy and safety of mechanical thrombectomy (MT) [3–5]. Nevertheless, after MT, approximately 54 % of patients are still unable to achieve functional independence within 3 months, and approximately 15 % of patients die within this timespan [6, 7]. These data show that factors other than recanalization status also play an important role in the prognosis of MT.

Body temperature (BT) is an important index and modifier of pathophysiologic events in AIS, and fever is a common symptom in AIS patients [8]. Hypothermia is considered to have a neuroprotective effect and to be a potential treatment in cerebral ischemia. Animal studies suggest that mild hypothermia therapy could reduce inflammation in brain tissue and improve outcomes after ischemic stroke [9]. However, a multicenter RCT showed that surface mild hypothermic treatment did not benefit AIS patients because of complications associated with hypothermia, such as infectious pneumonia. Geurts also pointed out that the effects of recanalization factors on patient outcomes were not considered, and the effects of mild hypothermia therapy are different in patients with different recanalization statuses of large intracranial vessels, such as non-recanalization and successful recanalization [10]. Although a number of studies have already proven the correlation between BT and clinical outcomes in AIS patients treated with intravenous thrombolysis [11, 12], studies evaluating the effect of BT levels on the outcomes of patients treated with MT are scarce. Considering that the successful recanalization rate with MT is higher than that with intravenous thrombolysis [13, 14] and that a certain incidence of re-occlusion still occurs after MT, it is necessary to conduct a study based on different recanalization statuses of patients.

The aim of the study was to determine whether BT levels have different effects on poststroke recovery in patients treated with MT during the first 3 months poststroke, especially in those with or without successful recanalization. It was hypothesized that hyperpyrexia would be associated with poorer clinical outcomes poststroke. Hence, we conducted this retrospective study on the effect of BT values on functional and survival outcomes in AIS patients treated with MT, and subgroup analysis was performed for different recanalization statuses.

## Methods

### Study Design and Patient Selection

**Fig. 1:**
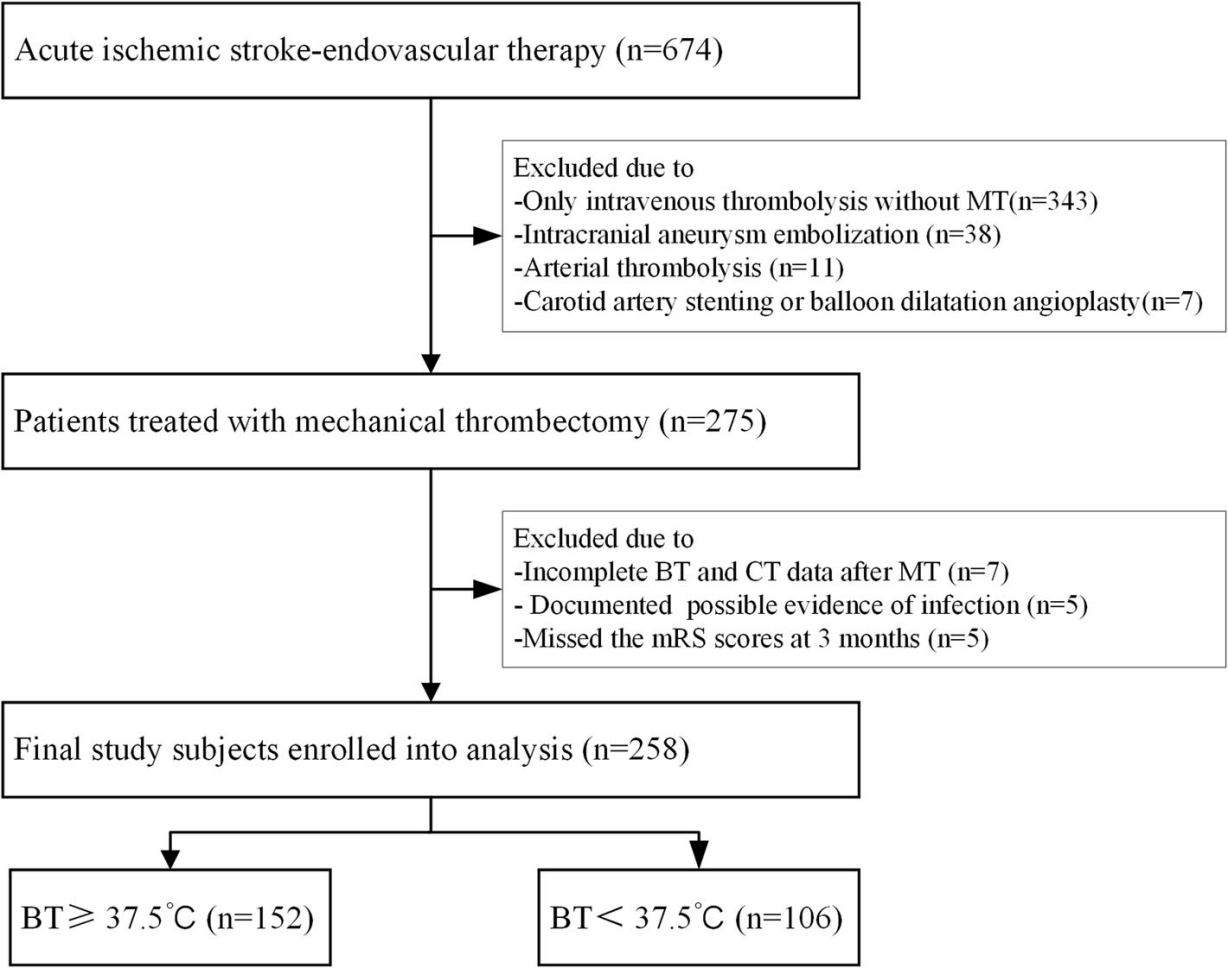
Flow chart of study cohort selection. BT, body temperature; mRS, modified Rankin Scale; MT, mechanical thrombectomy

We conducted a retrospective analysis of a hospital-based prospective database from September 2015 to September 2019 comprising patients (age ≥ 18 years) with acute ischemic stroke due to large vessel occlusion (LVO) who were treated with MT. The following patients were excluded: (1) patients with a pre-modified Rankin scale (mRS) score > 1; (2) patients with severe circulatory or respiratory failure or malignant tumor; (3) patients who could not fulfill the 3-month follow-up; and (4) patients with infection at admission. To exclude the influence of infection, we recorded documented evidence of infection at baseline, including physical examination, the white blood cell (WBC) count, procalcitonin level, the erythrocyte sedimentation rate, C-reactive protein (CRP), urinalysis, urine culture, and chest radiography, as in a previous study [15]. If any signs of infection were positive, we excluded the patient (Fig. 1). Patients underwent a nonenhanced computerized tomography (CT) head scan to exclude hemorrhage and MR imaging or CT angiography (CTA) to establish the arterial territory using standard criteria [16, 17]. There were no restrictions on neurological severity. The protocol was approved by the Ethics Committee of West China Hospital, Sichuan University.

### Temperature Measurement

The baseline BT was defined as the body temperature at admission. Temperatures were measured by an infrared ear thermometer upon admission and every 2 h for 24 h after MT. The peak BT value was defined as the highest value within 24 h, and we defined fever as a BT ≥ 37.5℃ [18]. Patients were divided into two groups according to whether they had a fever within 24 h.

### Data Collection and Definition

For each patient, we recorded the pre-stroke functional status using the mRS score and the history of stroke risk factors: systolic and diastolic blood pressure (SBP and DBP); the presence of diabetes mellitus (DM); atrial fibrillation and smoking. Vital signs and laboratory parameters at baseline before MT, the stroke cause according to the Trial of ORG 10,172 in Acute Stroke Treatment (TOAST) classification, the length of stay, intravenous thrombolysis, infection and/or antibiotic use were also recorded. Neurologists determined the National Institute of Health Stroke Scale (NIHSS) scores at admission, 24 h after MT and discharge to assess changes in stroke severity. The Alberta Stroke Program Early CT Score (ASPECTs) was used to assess the size of the baseline infarct size [19].

Functional outcomes were assessed by mRS scores at 3 months after the onset of symptoms. An mRS score of 0–2 was defined as a favorable outcome (functional independence), and a score of 0–1 was defined as an excellent outcome. The mRS scores were assessed by outpatient follow-up or telephone follow-up at 3 months. We obtained anesthesia type (general anesthesia or local anesthesia), procedure time, number of devices passes, and thrombolysis in cerebral infarction (TICI) scores to grade the degree of reperfusion from the reports of specialists at the end of MT. A TICI score of 2b-3 was defined as successful recanalization, and a score of 3 was defined as complete recanalization [20]. Symptomatic intracranial hemorrhage (sICH) was defined as any intraparenchymal, subarachnoid, or intraventricular hemorrhage on post-procedural CT that was associated with a ≥ 4-point increase in the NIHSS score according to the ECASS criteria [21]. HT was defined according to the ECASS criteria. HI1 was defined as a small petechiae hemorrhagic lesion along the margins of the infarct, and HI2 was defined as the internal fusion of the infarct into a patch of bleeding in the absence of a space-occupying effect. PH1 was defined as a hematoma area ≤ 30 % with a slight space-occupying effect, and PH2 was a hematoma area > 30 % with a significant space-occupying effect or hemorrhage in the remote infarcted area [21]. Early clinical improvement was defined as an improvement of ≥ 4 points in the NIHSS score at 24 h after onset compared with the score at admission or the disappearance of neurological function impairment (NIHSS 0–1) [22]. Early neurological deterioration was defined as an increase of ≥ 4 points in the total NIHSS score compared with the baseline value [23].

### Outcome Evaluation

Outcomes were evaluated in two ways. The primary outcomes included favorable outcomes and excellent outcomes. Mortality in the hospital and at 3 months, intracranial hemorrhage transformation (HT), sICH, early clinical improvement, and early neurological deterioration represented the secondary outcomes.

### Statistical Analysis

Continuous variables were summarized as the mean ± SD (normal distribution) or as the median with interquartile range (IQR) (non-normal distribution) and compared using an independent-samples *t* test, Mann-Whitney U test, or the non-parametric Kruskal-Wallis H test, as indicated. We described the categorical variables as numbers (n) with a percentage (%) and analyzed the significant differences between 2 and 3 groups by the χ^2^ test or Fisher’s exact test in the case of small, expected frequencies.

We constructed multivariable logistic regression models to assess the odds ratios (ORs) and corresponding 95 % CIs to evaluate the association between BT levels and 3-month functional outcomes, in-hospital and 3-month mortality, HT, sICH, early clinical improvement, and early neurological deterioration before and after adjusting for the following potential confounders based on their clinical significance and prior studies: age, sex, length of stay, NIHSS score at baseline, atrial fibrillation, smoking, serum glucose level, SBP, WBC, procedure time, successful recanalization and number of devices passes. We used the Bonferroni correction method to assess the primary and secondary outcomes, with a *P*-value < 0.05/number of comparisons as the threshold for statistical significance [24].

Subgroup analysis for heterogeneity of the BT effect was performed, with subgroups defined according to age, NIHSS score at baseline, number of devices passes, thrombolysis before MT, and successful recanalization. Moreover, we further analyzed the effect of BT levels on different recanalization statuses after MT and survival rates. Data analysis was performed with IBM SPSS version 25.0 (IBM corporation, Armonk, NY) and R software version 3.6.2 (R Foundation for Statistical Computing, Vienna, Austria). All *P* values presented were two-sided, and statistical significance was accepted if *P* < 0.05.

## Results

### Baseline characteristics

**Fig. 2:**
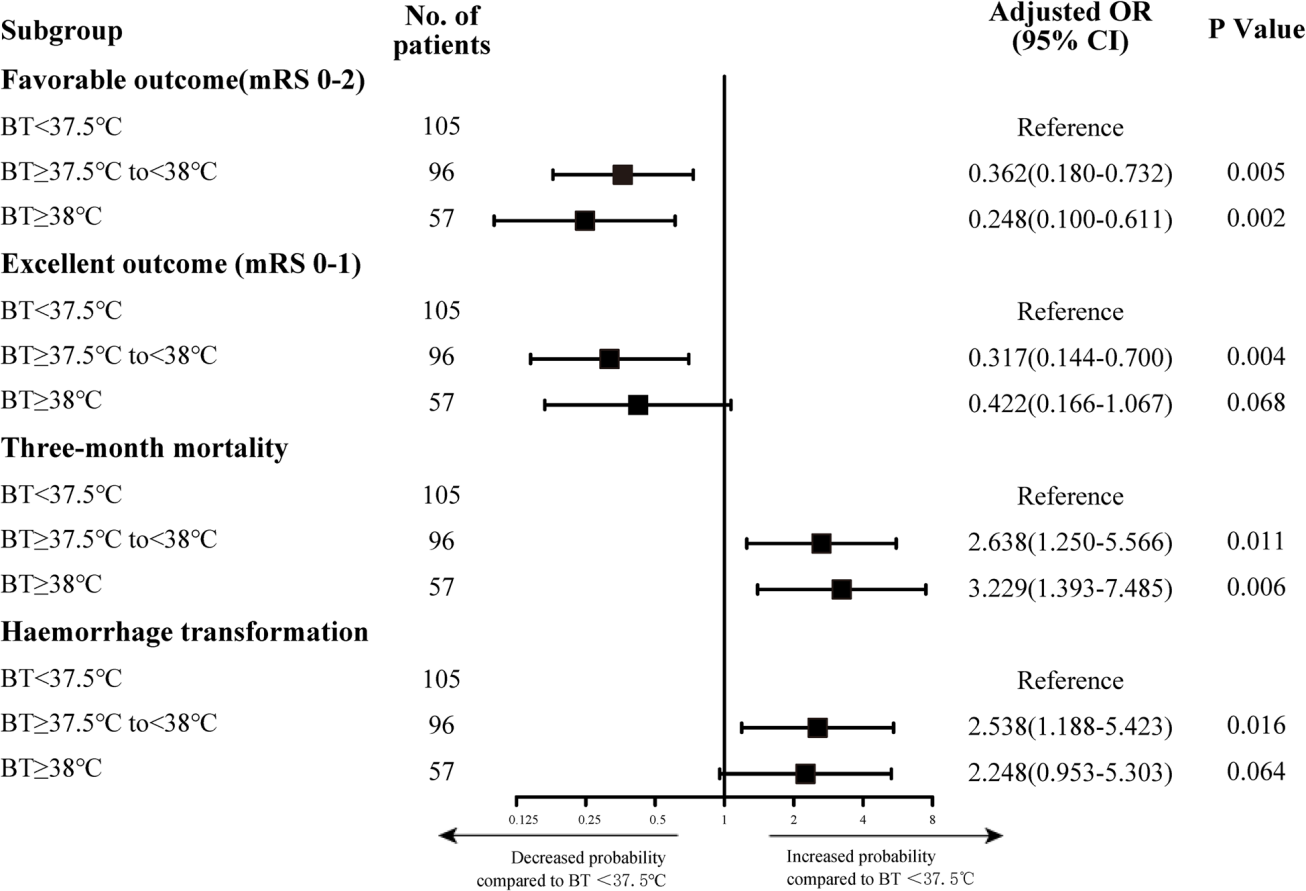
Sensitivity analysis of primary outcomes. The forest plot shows the differences in odds ratios for favorable outcomes (defined as mRS score of 0–2) at 3 months in the subgroups. Adjusted variables: age, sex, length of stay, NIHSS score at baseline, atrial fibrillation, smoking, serum glucose level, SBP, WBC, procedure time, successful recanalization and number of devices passes. mRS, modified Rankin Scales; NIHSS, National Institutes of Health Stroke Scale; MT, mechanical thrombectomy

In total, 258 consecutive patients (mean age 66.6 ± 14.4 years, 57.4 % men) were enrolled in the final study analysis (Fig. 2). The median NIHSS score was 17 points [IQR 13–22]. Of these cases, 249 (96.5 %) were treated within 6 h of symptom onset. The number of patients treated with MT more than 6 h after onset was 9 (3.5 %), with an average of 10.2 h. The associations between the peak BT levels within 24 h and the clinical characteristics are summarized in Table 1. Among 258 patients, 67 patients had internal carotid artery occlusions (26.0 %), 134 had middle cerebral artery occlusions (51.9 %), and 20 patients had tandem occlusions (7.8 %). The remaining patients had posterior circulation occlusions (14.3 %).

**Table 1:** Patients characteristics

**Parameter **	**All patients**	**BT <37.5℃**	**BT ≥37.5℃**	*P *value^*^
	** (*n*=258)**	**(*n*=106)**	**(*n*=152)**	
Demographic characteristics				
Age, mean (SD)	66.6(14.4)	66.7(14.0)	66.6(14.7)	0.960
Sex, male, n (%)	148(57.4)	64(60.4)	84(55.3)	0.414
History of risk factors, n (%)				
Hypertension	133(51.6)	58(54.7)	75(49.3)	0.395
Diabetes mellitus	68(26.4)	26(24.5)	42(27.6)	0.578
Smoking	85(32.9)	33(31.1)	52(34.2)	0.605
Drinking	63(24.4)	25(23.6)	38(25.0)	0.795
Dyslipidemia	16(6.2)	4(3.8)	12(7.9)	0.177
Previous stroke or TIA	37(14.3)	13(12.3)	24(15.8)	0.427
Atrial fibrillation	142(55.0)	63(59.4)	79(52.0)	0.236
Vital signs and laboratory parameters at baseline, mean (SD)				
Systolic blood pressure, mm Hg	142.0(26.0)	141.6(25.5)	142.2(26.4)	0.868
Diastolic blood pressure, mm Hg	81.8(13.9)	81.1(12.8)	82.3(14.8)	0.489
Serum glucose, mmol/L	8.3(3.0)	7.8(2.2)	8.6(3.5)	0.052
Total cholesterol, mmol/L	4.1(1.0)	4.0(1.0)	4.2(1.0)	0.097
LDL-C, mmol/L	2.4(0.8)	2.4(0.8)	2.5(0.8)	0.382
Triglyceride, mmol/L	1.5(1.0)	1.4(0.8)	1.6(1.2)	0.095
HDL-C, mmol/L	1.3(0.4)	1.2(0.4)	1.3(0.4)	0.106
Creatinine, μmol/L	78.0(25.5)	81.1(27.7)	75.8(23.6)	0.097
Platelet count, ×10^9^/μL	172.1(66.6)	173.9(73.1)	170.8(62.0)	0.723
White blood cell count, ×10^9^/μL	8.3(3.0)	8.2(3.0)	9.1(3.4)	0.043
Pre-MT BT (℃)	36.4(0.27)	36.5(0.24)	36.5(0.29)	0.188
Arterial territory, n (%)				0.091
ICA occlusion	67(26.0)	23(21.7)	44(28.9)	
MCA occlusion	134(51.9)	65(61.3)	69(45.4)	
Tandem occlusion	20(7.8)	6(5.7)	14(9.2)	
Posterior circulation occlusion	37(14.3)	12(11.3)	25(16.4)	
TOAST classification, n (%)				0.427
Large-artery atherosclerosis	93(36.0)	37(34.9)	56(36.8)	
Cardio-embolism	132(51.2)	52(49.1)	80(52.6)	
Undetermined etiology	33(12.8)	17(16.0)	16(10.5)	
Anesthesia type, n (%)				0.241
General anesthesia	222(86.0)	88(83.0)	134(88.2)	
Local anesthesia	36(14.0)	18(17.0)	18(11.8)	
Procedure time, min, mean (SD)	98.3(44.0)	90.7(37.3)	103.6(47.5)	0.020
NIHSS score at baseline, median (IQR)	17(13-22)	11(13-23)	16(11-20)	0.014
ASPECTS, median (IQR)	9(7-10)	9(8-10)	8(7-10)	0.157
Length of stay, day, median (IQR)	11(6-17)	16(11-20)	11(4-18)	0.600
Number of devices passes, mean (SD)	2.5(1.5)	2.4(1.5)	2.7(1.5)	0.086
Successful recanalization^a^, n (%)	191(74.0)	88(83.0)	103(67.8)	0.006
Intravenous thrombolysis, n (%)	70(27.1)	33(31.1)	37(24.3)	0.228

Values were measured for the peak body temperature within 24 h following mechanical thrombectomy

Abbreviations: *TIA* transient ischemic attack; *LDL-C* low-density lipoprotein cholesterol; *HDL-C* high-density lipoprotein cholesterol; *ICA* internal carotid artery; *MCA* middle cerebral artery; *TOAST* trial of ORG 10,172 in acute stroke treatment; *NIHSS* National Institutes of Health Stroke Scale; *TICI* thrombolysis in cerebral infarction; *ASPECs* Alberta Stroke Program Early CT Score; *MT* mechanical thrombectomy

^*^Continuous variables were compared between groups using independent samples *t* tests, Mann-Whitney U tests, or Kruskal-Wallis H tests. Categorical variables were analyzed by χ^2^ test, or Fisher’s exact tests as appropriate

^a^Successful recanalization indicates the TICI score of 2b-3

In our study, 7 patients (2.7 %) underwent MT using a combination of distal aspiration and stent retrieval, 5 patients (1.9 %) were treated with the distal aspiration technique, and 246 patients (95.3 %) underwent stent retrieval without distal aspiration, which was the most common technique. Seventy (27.1 %) patients underwent intravenous thrombolysis before MT. Successful recanalization was achieved in 74.0 % of the study population (n = 191). After excluding the patients with clinical evidence diagnosed as infection, there were only 3 patients whose BT was ≥ 37.5℃, of which two cases were 37.5℃ and one case was 38.4℃ on admission. Within 24 h after MT, the median maximum BT was 37.6 °C. According to BT levels within 24 h post MT, patients were divided into a fever group (n = 152; 58.9 %) and a non-fever group (n = 106; 41.1 %). Compared with the non-fever group, the fever group had higher baseline NIHSS scores (*P* = 0.014), lower rates of successful recanalization (*P* = 0.006), higher WBC counts (*P* = 0.043) and longer procedure times (*P* = 0.020). There were no differences in terms of the pre-MT BT, arterial territory, or infection post-MT between the two groups.

### Association between the post-MT body temperature and clinical outcomes

Table 2 showed the peak BT levels within 24 h after MT with different primary and secondary outcomes. Functional independence (mRS score of 0–2; favorable outcome) was confirmed in 83 (32.2 %) patients (Table 2, Additional file 1: Figure S1). The proportion of patients with functional independence was significantly lower in the fever group (23.0 % versus 45.3 %; *P* < 0.001 in the χ^2^ test). Similarly, the number of patients with excellent outcomes (mRS score of 0–1) was also lower in the fever group (13.8 % versus 31.1 %; *P* = 0.001 in the χ^2^ test; Table 2).

**Table 2:** Association between high body temperature levels (BT ≥ 37.5℃) and outcomes

Clinical Outcomes	BT < 37.5℃	BT ≥ 37.5℃		Crude OR (95 %CI)	*P* value	Adjusted OR^†^ (95 %CI)	*P* value^‡^
	**(*n* = 106), n (%)**		**(*n* = 152), n (%)**				
Primary outcomes at 3 months							
mRS,0–2	48(45.3)	35(23.0)		0.361 (0.211–0.619)	< 0.001	0.384 (0.201–0.733)	0.004
mRS,0–1	33(31.1)	21(13.8)		0.355 (0.191–0.658)	0.001	0.404 (0.200-0.817)	0.012
Secondary outcomes							
In-hospital mortality	5(4.7)	24(15.8)		3.787 (1.396–10.277)	0.009	2.796 (0.910–8.593)	0.073
Three-month mortality	18(17.0)	61(40.1)		3.277 (1.795–5.983)	< 0.001	3.087 (1.552–6.135)	0.001
HT	43(40.6)	73(48.0)		1.354 (0.820–2.236)	0.236	1.275 (0.746–2.178)	0.375
HI1	8(7.5)	5(3.3)					
HI2	6(5.7)	11(7.2)					
PH1	5(4.7)	6(3.9)					
PH2	21(19.8)	44(28.9)					
SAH/remote HT	3(2.8)	7(4.6)					
sICH	15(14.2)	41(27.0)		2.241 (1.166–4.306)	0.015	2.357 (1.176–4.723)	0.016
Early clinical improvement	62(58.2)	41(27.0)		0.262 (0.155–0.444)	< 0.001	0.260 (0.146–0.464)	< 0.001
Early neurological deterioration	13(12.3)	59(38.8)		4.538 (2.332–8.832)	< 0.001	4.780 (2.341–9.871)	< 0.001

Values were measured for the peak body temperature within 24 h following mechanical thrombectomy

Abbreviations: *BT* body temperature; *mRS* modified Rankin Scale; *OR* odds ratio; *HT* hemorrhage transformation; *HI* hemorrhagic infarction; *SAH* subarachnoid hemorrhage; *sICH* symptomatic intracranial hemorrhage

†The multiple logistic regression test was used to analyze ORs. Adjusted variables: age, sex, length of stay, NIHSS score at baseline, atrial fibrillation, smoking, serum glucose level, SBP, WBC, procedure time, successful reperfusion and number of devices passes

‡The Bonferroni correction method was used to assess the primary and secondary outcomes, and a *P* value < 0.05/number of comparisons was used as the threshold for statistical significance (*P* < 0.025 for primary outcomes and *P* < 0.008 for secondary outcomes)

Regarding secondary outcomes, 3-month mortality occurred more often in the fever group (40.1 % versus 17.0 %; *P* < 0.001 in the χ^2^ test). Despite similar rates of HT in the two groups (48.0 % versus 40.6 %; *P* = 0.239 in the χ^2^ test), the frequency of sICH was higher in the fever group (27.0 % versus 14.2 %; *P* < 0.001). The frequency of early clinical improvement was significantly higher in the non-fever group (58.2 % versus 27.0 %; *P* < 0.001), and early neurological deterioration was significantly lower in the non-fever group than in the fever group (12.3 % versus 38.8 %; *P* < 0.001). No difference was noted in the BT level among patients with HT (*P* = 0.236; Table 2).

In the multivariable analysis, we included adjustments for confounders, and higher BT levels showed a negative correlation with favorable outcomes (OR, 0.384; 95 % CI, 0.201–0.733; *P* = 0.004) and excellent outcomes (OR, 0.404; 95 % CI, 0.200-0.817; *P* = 0.012; Table 2). Higher BT levels were also significantly associated with increased 3-month mortality (OR, 3.087; 95 % CI, 1.552–6.135; *P* = 0.001), sICH (OR, 2.357;95 % CI, 1.176–4.723; *P* = 0.016), and early neurological deterioration (OR, 4.780; 95 % CI, 2.341–9.871; *P* < 0.001). However, early clinical improvement was negatively associated with higher BT levels (OR, 0.260; 95 % CI, 0.146–0.464; *P* < 0.001). In-hospital mortality did not differ between the two groups in the multivariable analysis (*P* = 0.073).

### Subgroups analyzed the predictive value of the body temperature for clinical outcomes post-MT

As hyperpyrexia was an independent predictor of poststroke outcomes, we further performed a sensitivity analysis, which was based on different BT levels. BT ≥ 37.5℃ was negatively correlated with favourable outcomes at 3 months compared with the reference group with BT < 37.5℃, irrespective of the precise BT level (Fig. 2). However, there was no statistically significant difference when BT was ≥ 38℃ in terms of excellent outcomes or the rate of HT. Higher BT levels, especially BT ≥ 38℃, were associated with a significantly increased risk of mortality at 3 months (OR, 3.229; 95 % CI, 1.393–7.485).

**Fig. 3:**
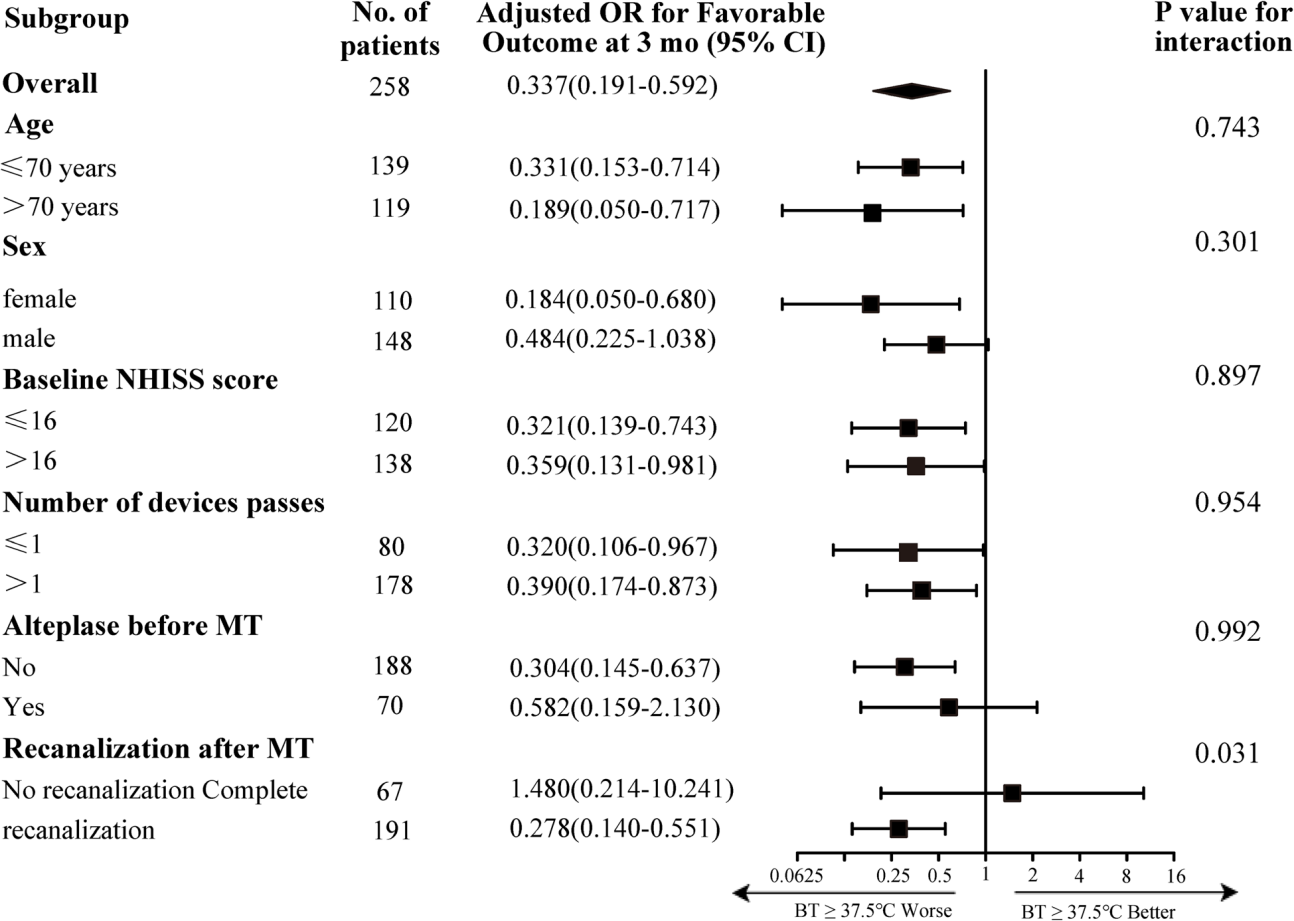
Subgroup analysis of body temperature levels with clinical outcomes post-mechanical thrombectomy. Adjusted variables: age, sex, length of stay, NIHSS score at baseline, atrial fibrillation, smoking, serum glucose level, SBP, WBC, procedure time, successful recanalization and number of devices passes. mRS, modified Rankin Scales; NIHSS, National Institutes of Health Stroke Scale

In the subgroup analysis, there was significant heterogeneity in the ORs for favorable outcomes associated with fever patients who had successful recanalization post-MT and those who did not (Fig. 3). There was no evidence of heterogeneity in the effect of BT levels due to other prespecified variables, such as the number of the devices passes and intravenous thrombolysis before MT.

Based on the above results, we further performed a subgroup analysis of BT levels between patients with good and poor outcomes according to the achieved recanalization status (Table 3). The results showed that patients with favorable outcomes had significantly lower BT levels than those with poor outcomes. Moreover, the difference was significant in patients with successful recanalization (TICI 2b-3) and complete recanalization (TICI 3). Additional file 2: Figure S2 showed the Kaplan-Meier analysis of patient survival rates. The non-fever group had significantly better survival rates than the fever group within 24 h after MT (*P* < 0.001; log-rank test).

**Table.3:** Comparison of different BT levels in subgroup analysis according to the achieved recanalization status

	TICI < 2b			TICI ≥ 2b			TICI = 3		
**Patient group**	**mRS 0–2**	**mRS 3–6**	***P*value**	**mRS 0–2**	**mRS 3–6**	***P*value**	**mRS 0–2**	**mRS 3–6**	***P*value**
N	11	56		79	112		62	95	
Pre-MT BT (℃)	36.5(0.13)	36.5(0.36)	0.875	36.5(0.24)	36.5(0.25)	0.736	36.5(0.25)	36.5(0.24)	0.947
6 h BT (℃)	37.0(0.60)	37.2(0.94)	0.677	36.7(0.39)	37.0(0.78)	0.058	36.7(0.34)	37.0(0.76)	0.026
12 h BT (℃)	37.1(0.64)	37.2(0.74)	0.709	36.8(0.41)	37.2(0.81)	< 0.001	36.7(0.37)	37.2(0.78)	< 0.001
24 h BT (℃)	37.1(0.58)	37.3(0.60)	0.319	36.9(0.54)	37.3(0.71)	< 0.001	36.8(0.53)	37.3(0.69)	< 0.001
Peak 24-hour BT (℃)	37.9(0.46)	37.8(0.68)	0.753	37.3(0.51)	37.7(0.65)	< 0.001	37.2(0.49)	37.7(0.61)	< 0.001

All values were expressed as the mean ± SD

Abbreviations: *BT* body temperature; *N* number; *TICI* thrombolysis in cerebral infarction; *mRS* modified Rankin Scale

### Body temperature, infection/antibiotic use, and 90-day clinical outcomes after MT

Besides, more than 50 % of patients who underwent MT developed fever within 24 h. Given the close link between fever and infectious disease, we analyzed the incidence of infection/antibiotic use within 24 h and over 24 h after MT (Additional file 3: Table S1) and investigated the association between the infection diagnosis /antibiotic use during hospitalization and clinical outcomes (Additional file 4: Table S2).

Fifty-one patients were diagnosed with infection/antibiotic use within 24 h after MT. A total of 105 patients received a diagnosis with infection/antibiotics over 24 h post-MT. The main types of infection were pneumonia and urinary tract infection. Piperacillin-tazobactam was the most used antibiotic. Among patients with a fever within 24 h post-MT who were not diagnosed with infection, 36.8 % were diagnosed with an infection over a further 24 h. Table S2 showed that infection within 24 h after MT had a significant effect on the favorable outcome (*P* = 0.032). On the contrary, infection beyond 24 h had no effect on the favorable outcome (*P* = 0.066).

## Discussion

Our results showed that increasing peak BT levels within 24 h post-MT were associated with a higher likelihood of 3-month mortality and lower odds of functional independence. These associations were independent of the age, sex, length of stay, NIHSS score at baseline, atrial fibrillation, smoking, serum glucose level, SBP, WBC, procedure time, successful recanalization, and number of the devices passes. The chance of a good outcome was halved, and the risk of death was increased four-fold when BT was ≥ 38℃. However, there was no association between the BT level and the risk of HT. Currently, there are few studies on the relationship between BT levels and clinical prognosis post-MT, especially the effects on recanalization statuses and survival rates. Therefore, this study may provide a basis for future studies on the correlation of fever post-MT and clinical outcomes.

By previous studies, hyperpyrexia has an adverse effect on functional outcomes in patients treated with tissue-type plasminogen activator (t-PA) [25, 26]. However, the potential adverse effects of fever associated with early reperfusion can be better studied after MT, since the successful reperfusion rate of MT is higher than that of intravenous t-PA [13, 14]. Another study reported that patients with a higher baseline BT had less severe neurological deficits and smaller infarct volumes [11]. Part of our results were in accordance with a recent study on the association between BT and outcomes in patients treated with MT, and researchers believed that increased BT both before and after MT worsened the prognosis of patients [27]. In contrast to the referenced study [27], our results showed a few differences. First, in the present study, when excluding patients with acute infection at admission, we found that pre-MT BT levels did not affect functional outcomes and survival rates. The vast majority of patients in our study had normal baseline BT levels on admission. Furthermore, we excluded patients with an acute infection on admission, which may have led to the differences from the previous study. Second, our results showed that upon a priori dichotomizing all patients according to the definite recanalization status (TICI of 0-2b versus TICI of 2b-3), hyperpyrexia had a greater impact on successful recanalization patients treated with MT than those who did not, demonstrating the need for individualized treatment of patients with different recanalization statuses. However, the underlying mechanisms are unclear and need to be further studied.

A preclinical study has shown that hypothermia therapy confers greater protective effects [28]. However, clinical trials have failed to demonstrate the benefit of therapeutic hypothermia. The Cooling for Ischemic Stroke Trial (COOLIST) was a multicenter randomized controlled trial that showed that surface cooling is not feasible below 35 °C, and mild hypothermia could lead to pneumonia, which offset the benefits of hypothermia therapy [10]. Therefore, maintenance of a normal BT is more feasible and safer than hypothermia treatment. Also, this study showed different effects on functional outcomes according to BT levels. In the subgroup analysis, an association between lower BT levels and better clinical outcomes was observed in the successful recanalization group (*P* < 0.001) but not in the non-recanalization group. Thus, our results support that further investigation of early BT control in patients with hyperpyrexia, especially patients with successful recanalization and BT ≥ 37.5℃, is warranted. Future research should investigate the association between early hypothermia therapy and infarct volumes and whether mild hypothermia benefits poststroke recovery in patients with successful recanalization undergoing endovascular therapy.

In this study, more than half of the patients who underwent MT developed a fever. Previous studies have demonstrated that age, stroke severity, stroke type, lesion volume, infection, and inflammatory response may be determinants of fever after AIS [29–31]. Other causes are hospital-acquired infections and complications such as deep vein thrombosis [32]. Given the strong relationship between fever and infection, we analyzed the relationships between fever, infection/antibiotic use at different time points and clinical outcomes at 90 days after AIS. The results showed that 156 patients (60.4 %) were diagnosed with the infection. Infection within 24 h after MT was associated with poor 3-month outcome. However, infection was not a factor that affected favorable outcome 90 days over 24 h after MT. These findings lend further support to the hypothesized detrimental influence of temperature elevation on the fate of ischemic tissues in the early aftermath of cerebrovascular injury, which were consistent with the research results of Nowak et al. [33]. Several recent clinical trials tested preventive antibiotic therapy could reduce the occurrence of post-AIS infection and provided additional benefits over standard treatment [34, 35]. Therefore, when necessary, prophylactic use of antibiotics should be given to patients at risk of infection to reduce the incidence of post-MT infection. Patients under general anesthesia have a higher risk of infection after endovascular treatment [36, 37]. Thus, for patients who can protect their airway and are cooperative, local anesthesia with conscious sedation of AIS are feasible to short anesthesia time, operation time, which can reduce the chance of iatrogenic infections during MT. More importantly, using comprehensive treatment measures to maintain normal BT, rather than hyperthermia or hypothermia treatment, is more beneficial to patients’ long-term clinical outcomes.

There are some limitations in this study. First, we evaluated the effect of BT on outcomes for a limited duration of time, and the impact of BT during MT and BT variability were not assessed in the study, although we analyzed the effects of pre-MT and post-MT body temperature levels. Second, only patients who had undergone MT were included, without considering those who underwent other types of procedures, such as arterial stenting or balloon dilatation angioplasty. In addition, although we excluded patients with possible infection and patients with severe circulatory or respiratory failure or malignant tumors at admission, the effects of drug factors on BT were not fully assessed. Additionally, although we adjusted for possible confounding variables, several risk factors, such as physical activity, socioeconomic status was not analyzed. Finally, we did not estimate the final infarct size. It was proven that a higher BT at 24 h after stroke was associated with a greater hypodensity volume and worse outcomes in AIS patients treated with thrombolytic therapy [25]. Exploring infarct sizes may provide an important basis for finding the cause of hyperthermia in patients. Further large, well-designed studies including measurements of infarct size are required to better characterize the underlying relationships between the infarct size, BT levels, and clinical outcomes.

## Conclusions

In summary, our findings provide preliminary data on the relationship between post-MT hyperpyrexia and clinical outcomes, especially when BT ≥ 38℃. Post-MT hyperpyrexia is an independent predictor of 3-month functional independence and mortality and may augment the risk of sICH and early neurological deterioration, particularly in those with successful recanalization. Therefore, it is essential to assess the early temperature of post-MT patients for the risk of adverse clinical outcomes and mortality. The results encourage prospective studies to investigate the robust potential benefits of post-MT recovery in patients with lower BT levels.

## Supplementary Information

**Additional file 1: Supplementary Figure S1.** Distribution of modified Rankin Scale (mRS) scores at 3 months in each group (OR, 0.361; 95 %CI, 0.211–0.619; *P* < 0.001). BT, body temperature.

**Additional file 2: Supplementary Figure S2.** Kaplan-Meier survival curve at different time points compared between the fever group (BT ≥ 37.5℃) and non-fever group (BT < 37.5℃) at baseline (A), body temperatures 6 h post-MT (B), body temperatures 12 h post-MT(C), and peak body temperatures within 24 h post-MT(D). P-values were derived using the log-rank test.

**Additional file 3: Supplementary Table S1.** Distribution of BT at 24h post-MT with diagnosed infection/antibiotics use during the entire course of hospitalization.

**Additional file 4: Supplementary Table S2.** Relationships between diagnosis of infection/antibiotics use and clinical outcomes at different time points after MT.

## Data Availability

Data are available upon reasonable request.

## Abbreviations

AIS: Acute ischemic stroke
RCT: Randomized controlled clinical trial
MT: Mechanical thrombectomy
LVO: Large vessel occlusion
BT: Body temperature
mRS: Modified Rankin scale
WBC: White blood cell
CRP: C-reaction protein
CT: Computerized tomography
CTA: CT angiography
SBP: Systolic blood pressure
DBP: Diastolic blood pressure
DM: Diabetes mellitus
TOAST: Trial of ORG 10172 in Acute Stroke Treatment
NIHSS: National Institute of Health Stroke Scale
ASPECTs: Alberta Stroke Program Early CT Score
TICI: Thrombolysis in cerebral infarction
sICH: Symptomatic intracranial hemorrhage
HT: Hemorrhage transformation
HI: Hemorrhagic infarction
SAH: Subarachnoid hemorrhage
ORs: Odds ratios
CI: Confidence interval
IQR: Interquartile range
TIA: Transient ischemic attack
LDL-C: Low-density lipoprotein cholesterol
HDL-C: High-density lipoprotein cholesterol
ICA: Internal carotid artery
MCA: Middle cerebral artery
