# Correction to: Association between hyperpyrexia and poststroke outcomes in patients with recanalization after mechanical thrombectomy: a retrospective cohort study

**DOI:** 10.1186/s12883-021-02453-9

**Published:** 2021-11-19

**Authors:** Man Chen, Jinghuan Fang, Xintong Wu, Qin Liu, Ling Feng, Li He

**Affiliations:** 1 Department of Neurology, West China Hospital, Sichuan University, Chengdu, 610041 China; 2 Department of Neurology, West China Hospital, Sichuan University/West China School of Nursing, Sichuan University, Chengdu, 610041 China

**Correction to: BMC Neurol 21, 365 (2021)**

**https://doi.org/10.1186/s12883-021-02400-8**

Following publication of the original article [1], the authors reported errors in the table heading for Tables 1, 2 and 3 and Fig. 3.

**Table 1:** Patients characteristics

Parameter	All patients	BT <37.5°C	BT ≥37.5°C	***P*** value^*^
	(***n***=258)	(***n***=106)	(***n***=152)	
Demographic characteristics				
Age, mean (SD)	66.6(14.4)	66.7(14.0)	66.6(14.7)	0.960
Sex, male, n (%)	148(57.4)	64(60.4)	84(55.3)	0.414
History of risk factors, n (%)				
Hypertension	133(51.6)	58(54.7)	75(49.3)	0.395
Diabetes mellitus	68(26.4)	26(24.5)	42(27.6)	0.578
Smoking	85(32.9)	33(31.1)	52(34.2)	0.605
Drinking	63(24.4)	25(23.6)	38(25.0)	0.795
Dyslipidemia	16(6.2)	4(3.8)	12(7.9)	0.177
Previous stroke or TIA	37(14.3)	13(12.3)	24(15.8)	0.427
Atrial fibrillation	142(55.0)	63(59.4)	79(52.0)	0.236
Vital signs and laboratory parameters at baseline, mean (SD)				
Systolic blood pressure, mm Hg	142.0(26.0)	141.6(25.5)	142.2(26.4)	0.868
Diastolic blood pressure, mm Hg	81.8(13.9)	81.1(12.8)	82.3(14.8)	0.489
Serum glucose, mmol/L	8.3(3.0)	7.8(2.2)	8.6(3.5)	0.052
Total cholesterol, mmol/L	4.1(1.0)	4.0(1.0)	4.2(1.0)	0.097
LDL-C, mmol/L	2.4(0.8)	2.4(0.8)	2.5(0.8)	0.382
Triglyceride, mmol/L	1.5(1.0)	1.4(0.8)	1.6(1.2)	0.095
HDL-C, mmol/L	1.3(0.4)	1.2(0.4)	1.3(0.4)	0.106
Creatinine, μmol/L	78.0(25.5)	81.1(27.7)	75.8(23.6)	0.097
Platelet count, ×10^9^/μL	172.1(66.6)	173.9(73.1)	170.8(62.0)	0.723
White blood cell count, ×10^9^/μL	8.3(3.0)	8.2(3.0)	9.1(3.4)	0.043
Pre-MT BT (°C)	36.4(0.27)	36.5(0.24)	36.5(0.29)	0.188
Arterial territory, n (%)				0.091
ICA occlusion	67(26.0)	23(21.7)	44(28.9)	
MCA occlusion	134(51.9)	65(61.3)	69(45.4)	
Tandem occlusion	20(7.8)	6(5.7)	14(9.2)	
Posterior circulation occlusion	37(14.3)	12(11.3)	25(16.4)	
TOAST classification, n (%)				0.427
Large-artery atherosclerosis	93(36.0)	37(34.9)	56(36.8)	
Cardio-embolism	132(51.2)	52(49.1)	80(52.6)	
Undetermined etiology	33(12.8)	17(16.0)	16(10.5)	
Anesthesia type, n (%)				0.241
General anesthesia	222(86.0)	88(83.0)	134(88.2)	
Local anesthesia	36(14.0)	18(17.0)	18(11.8)	
Procedure time, min, mean (SD)	98.3(44.0)	90.7(37.3)	103.6(47.5)	0.020
NIHSS score at baseline, median (IQR)	17(13-22)	11(13-23)	16(11-20)	0.014
ASPECTS, median (IQR)	9(7-10)	9(8-10)	8(7-10)	0.157
Length of stay, day, median (IQR)	11(6-17)	16(11-20)	11(4-18)	0.600
Number of devices passes, mean (SD)	2.5(1.5)	2.4(1.5)	2.7(1.5)	0.086
Successful recanalization^a^, n (%)	191(74.0)	88(83.0)	103(67.8)	0.006
Intravenous thrombolysis, n (%)	70(27.1)	33(31.1)	37(24.3)	0.228

Values were measured for the peak body temperature within 24 hours following mechanical thrombectomy

*Abbreviations*: *TIA* transient ischemic attack, *LDL-C* low-density lipoprotein cholesterol, *HDL-C* high-density lipoprotein cholesterol, *ICA* internal carotid artery, *MCA* middle cerebral artery, *TOAST* trial of ORG 10172 in acute stroke treatment, *NIHSS* National Institutes of Health Stroke Scale, *TICI* thrombolysis in cerebral infarction, *ASPECs* Alberta Stroke Program Early CT Score, *MT* mechanical thrombectomy

^*^ Continuous variables were compared between groups using independent samples *t* tests, Mann-Whitney U tests, or Kruskal-Wallis H tests. Categorical variables were analyzed by χ^2^ test, or Fisher’s exact tests as appropriate

^a^ Successful recanalization indicates the TICI score of 2b-3

**Table 2:** Association between high body temperature levels (BT≥37.5°C) and outcomes

Clinical outcomes	BT <37.5°C	BT ≥37.5°C	Crude OR (95%CI)	***P*** value	Adjusted OR^**†**^ (95%CI)	***P*** value^**‡**^
	(***n***=106), n (%)	(***n***=152), n (%)				
Primary outcomes at 3 months						
mRS,0-2	48(45.3)	35(23.0)	0.361 (0.211-0.619)	<0.001	0.384 (0.201-0.733)	0.004
mRS,0-1	33(31.1)	21(13.8)	0.355 (0.191-0.658)	0.001	0.404 (0.200-0.817)	0.012
Secondary outcomes						
In-hospital mortality	5(4.7)	24(15.8)	3.787 (1.396-10.277)	0.009	2.796 (0.910-8.593)	0.073
Three-month mortality	18(17.0)	61(40.1)	3.277 (1.795-5.983)	<0.001	3.087 (1.552-6.135)	0.001
HT	43(40.6)	73(48.0)	1.354 (0.820-2.236)	0.236	1.275 (0.746-2.178)	0.375
HI1	8(7.5)	5(3.3)				
HI2	6(5.7)	11(7.2)				
PH1	5(4.7)	6(3.9)				
PH2	21(19.8)	44(28.9)				
SAH/remote HT	3(2.8)	7(4.6)				
sICH	15(14.2)	41(27.0)	2.241 (1.166-4.306)	0.015	2.357 (1.176-4.723)	0.016
Early clinical improvement	62(58.2)	41(27.0)	0.262 (0.155-0.444)	<0.001	0.260 (0.146-0.464)	<0.001
Early neurological deterioration	13(12.3)	59(38.8)	4.538 (2.332-8.832)	<0.001	4.780 (2.341-9.871)	<0.001

Values were measured for the peak body temperature within 24 hours following mechanical thrombectomy

*Abbreviations*: *BT* body temperature, *mRS* modified Rankin Scale, *OR* odds ratio, *HT* hemorrhage transformation, *HI* hemorrhagic infarction, *SAH* subarachnoid hemorrhage, *sICH* symptomatic intracranial hemorrhage

† The multiple logistic regression test was used to analyze ORs. Adjusted variables: age, sex, length of stay, NIHSS score at baseline, atrial fibrillation, smoking, serum glucose level, SBP, WBC, procedure time, successful reperfusion and number of devices passes

‡ The Bonferroni correction method was used to assess the primary and secondary outcomes, and a *P* value <0.05/number of comparisons was used as the threshold for statistical significance (*P*<0.025 for primary outcomes and *P*<0.008 for secondary outcomes)

**Table 3:** Comparison of different BT levels in subgroup analysis according to the achieved recanalization status

Patient group	TICI <2b			TICI ≥2b			TICI =3		
	mRS 0-2	mRS 3-6	***P*** value	mRS 0-2	mRS 3-6	***P*** value	mRS 0-2	mRS 3-6	***P*** value
N	11	56		79	112		62	95	
Pre-MT BT (°C)	36.5(0.13)	36.5(0.36)	0.875	36.5(0.24)	36.5(0.25)	0.736	36.5(0.25)	36.5(0.24)	0.947
6 hours BT (°C)	37.0(0.60)	37.2(0.94)	0.677	36.7(0.39)	37.0(0.78)	0.058	36.7(0.34)	37.0(0.76)	0.026
12 hours BT (°C)	37.1(0.64)	37.2(0.74)	0.709	36.8(0.41)	37.2(0.81)	<0.001	36.7(0.37)	37.2(0.78)	<0.001
24 hours BT (°C)	37.1(0.58)	37.3(0.60)	0.319	36.9(0.54)	37.3(0.71)	<0.001	36.8(0.53)	37.3(0.69)	<0.001
Peak 24-hour BT (°C)	37.9(0.46)	37.8(0.68)	0.753	37.3(0.51)	37.7(0.65)	<0.001	37.2(0.49)	37.7(0.61)	<0.001

All values were expressed as the mean(SD)

*Abbreviations*: *BT* body temperature, *N* number, *TICI* thrombolysis in cerebral infarction, *mRS* modified Rankin Scale

**Fig. 3:**
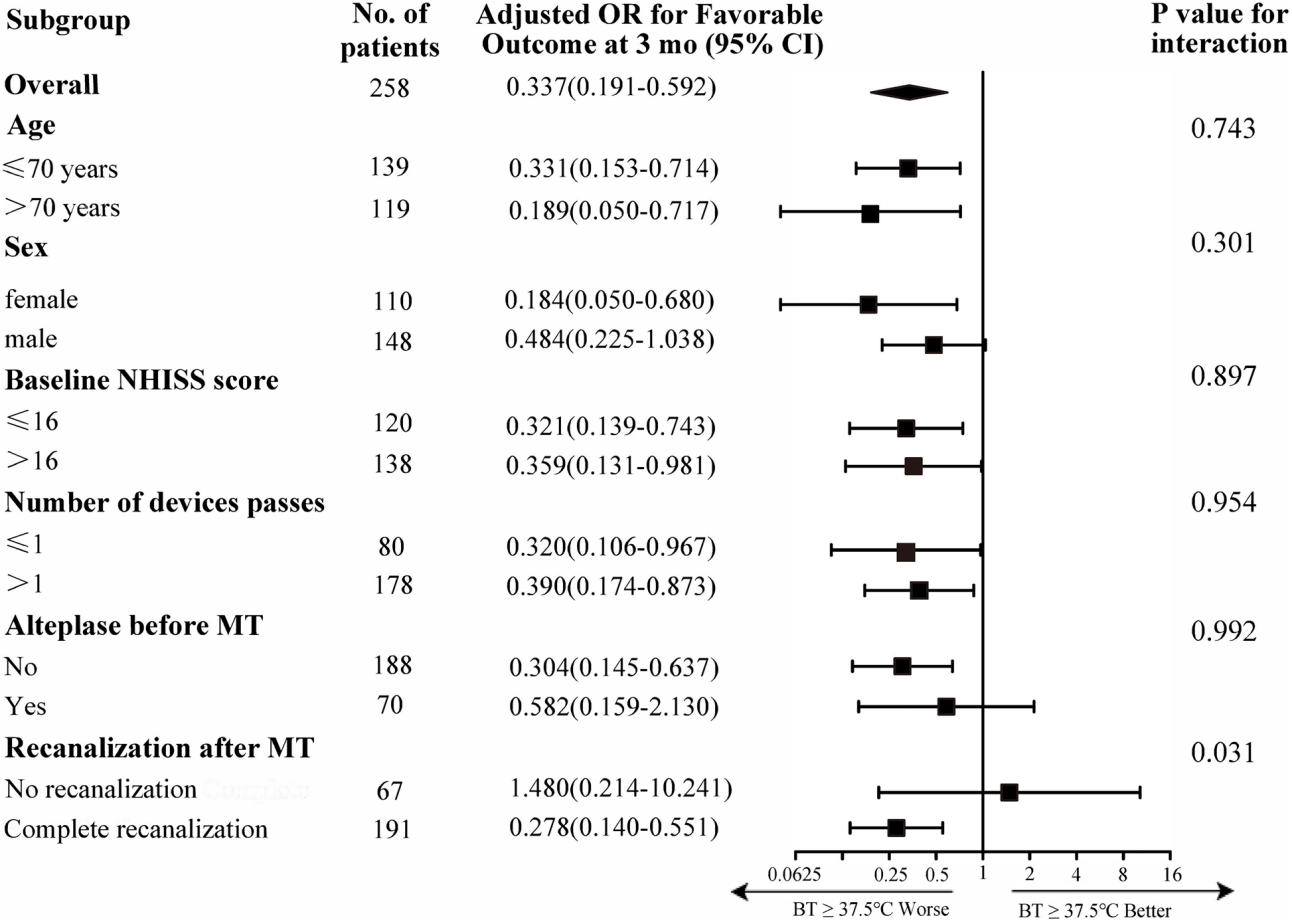
Subgroup analysis of body temperature levels with clinical outcomes post-mechanical thrombectomy. Adjusted variables: age, sex, length of stay, NIHSS score at baseline, atrial fibrillation, smoking, serum glucose level, SBP, WBC, procedure time, successful recanalization and number of devices passes. mRS, modified Rankin Scales; NIHSS, National Institutes of Health Stroke Scale

Table 1: There should only be two rows in the heading, “All patients”, “BT < 37.5°C” and “*P* value*” should be in a single row and a vertical line should cross below these headings (“All patients”, “BT < 37.5°C”, “BT ≥37.5°C”).

Table 2: Column 1, “Clinical Outcomes” should be changed to “Clinical outcomes”. Column 2, a vertical line should run across below “BT < 37.5°C” the same vertical line found in column 3. And keep “BT≥ 37.5°C” and “(*n*=152)” aligned up and down in column 3, the same for column 2.

Table 3: The second row for columns 2-3 and 5-6, there should be a vertical line that crosses above “mRS 0–2 and mRS 3–6” the same line found in columns 8 and 9.

Figure 3: The last section entry “No recanalization Complete recanalization” should be separated into two entries “No recanalization” and “Complete recanalization”.

The original article [1] has been updated.
