# Supplementary figures and images for: Association between hyperpyrexia and poststroke outcomes in patients with recanalization after mechanical thrombectomy: a retrospective cohort study
Source: BMC Neurol. 2021 Sep 21;21:365. doi: 10.1186/s12883-021-02400-8 (PMC8454168; doi:10.1186/s12883-021-02400-8)

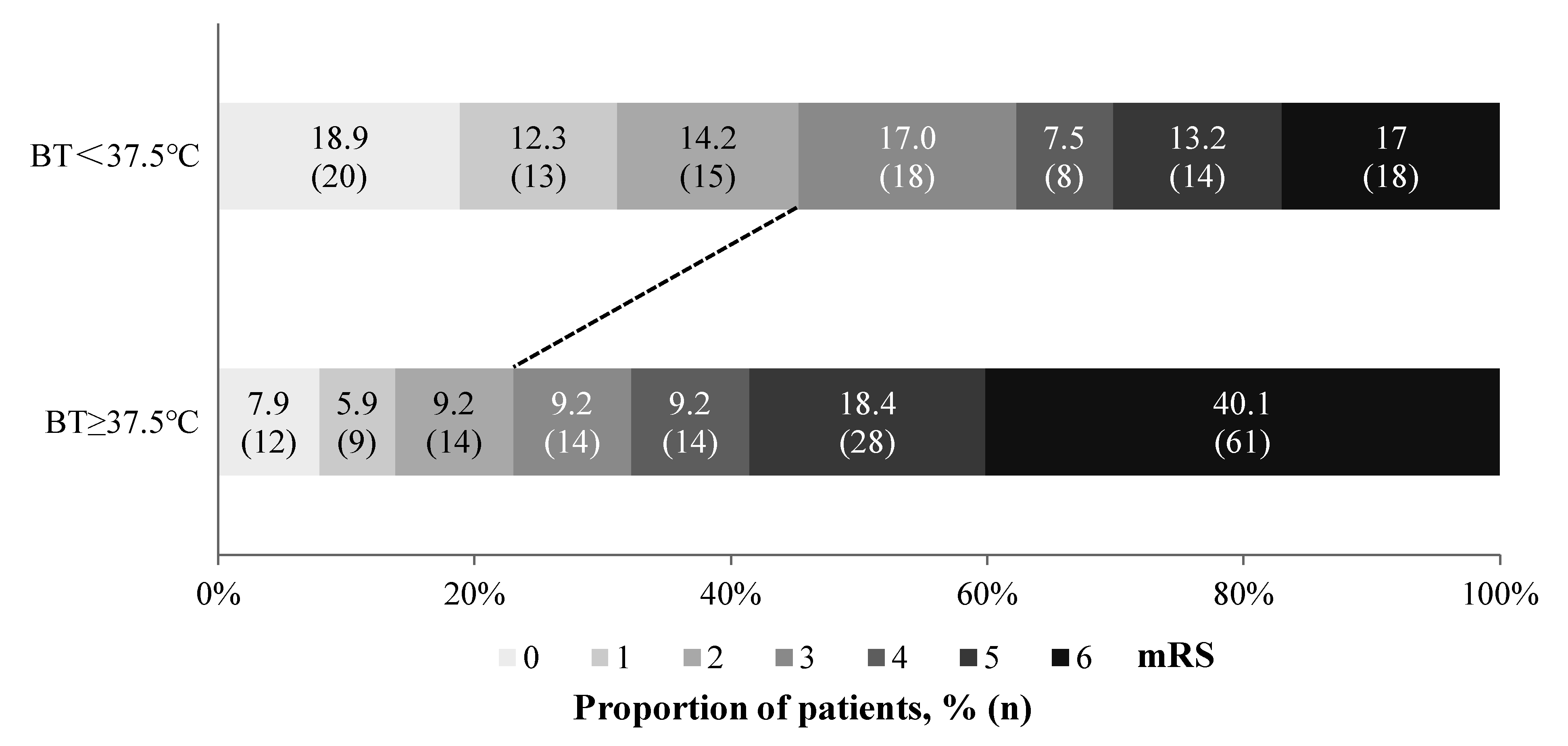

Supplement: Supplementary file 1 — Additional file 1: Supplementary Figure S1. Distribution of modified Rankin Scale (mRS) scores at 3 months in each group (OR, 0.361; 95 %CI, 0.211–0.619; P < 0.001). BT, body temperature. [file 12883_2021_2400_MOESM1_ESM.tiff]

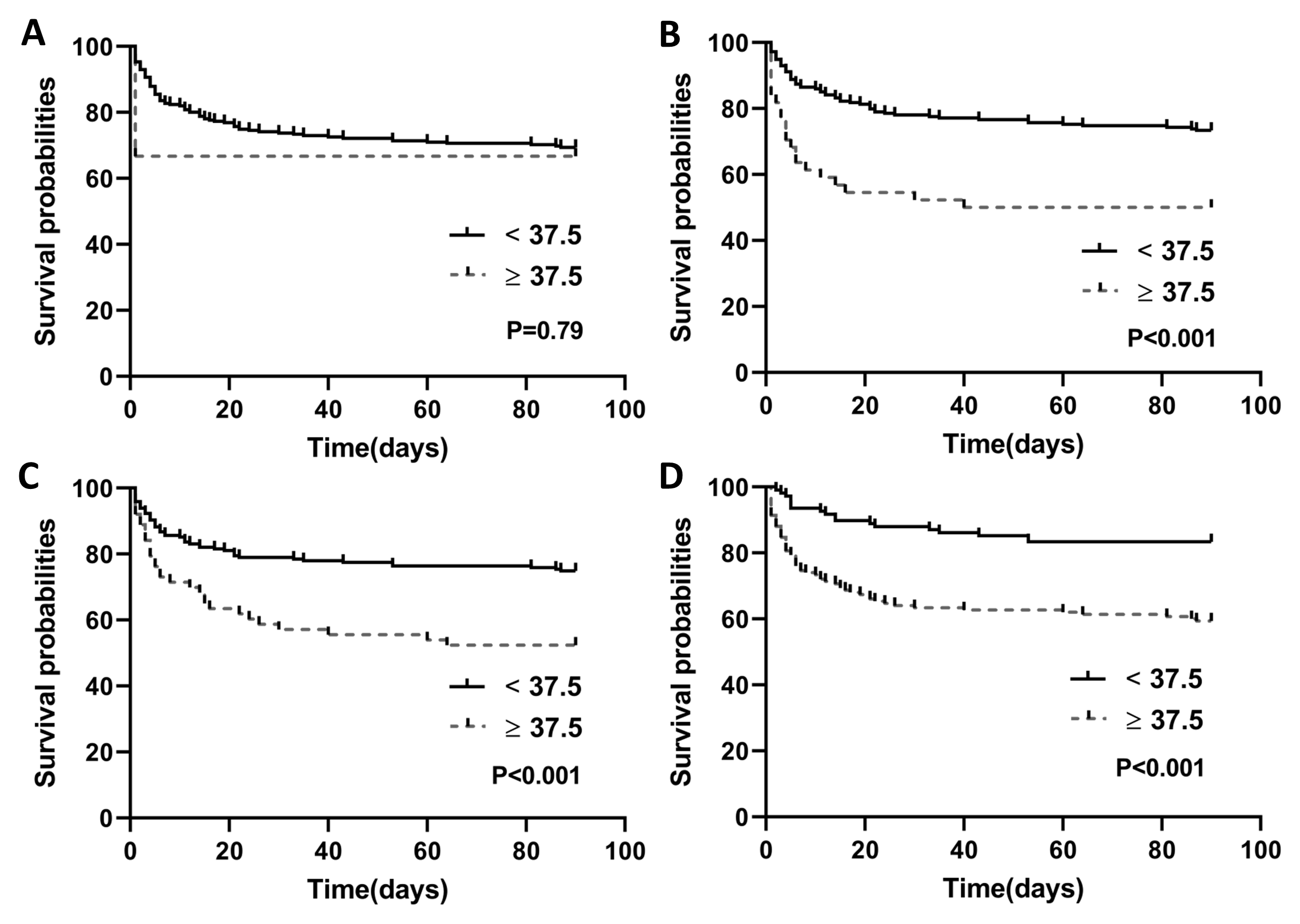

Supplement: Supplementary file 2 — Additional file 2: Supplementary Figure S2. Kaplan-Meier survival curve at different time points compared between the fever group (BT ≥ 37.5℃) and non-fever group (BT < 37.5℃) at baseline (A), body temperatures 6 h post-MT (B), body temperatures 12 h post-MT(C), and peak body temperatures within 24 h post-MT(D). P-values were derived using the log-rank test. [file 12883_2021_2400_MOESM2_ESM.tif]
