# Supplementary material for: Association between hyperpyrexia and poststroke outcomes in patients with recanalization after mechanical thrombectomy: a retrospective cohort study
Source: BMC Neurol. 2021 Sep 21;21:365. doi: 10.1186/s12883-021-02400-8 (PMC8454168; doi:10.1186/s12883-021-02400-8)
Supplement: Supplementary file 3 — Additional file 3: Supplementary Table S1. Distribution of BT at 24h post-MT with diagnosed infection/antibiotics use during the entire course of hospitalization. [file 12883_2021_2400_MOESM3_ESM.docx]

**Supplementary Table 1** Distribution of BT at 24h post-MT with diagnosed infection/antibiotics use during the entire course of hospitalization

| **Parameter** | **All patients** | **TB＜37.5℃** | **TB≥ 37.5℃** |
| --- | --- | --- | --- |
| Antibiotics use within 24 h after MT, n (%) | 51(19.8) | 2(1.9) | 49(32.2) |
| Piperacillin-tazobactam | 39 | 0 | 39 |
| Cephalosporin antibiotics | 6 | 1 | 5 |
| Moxifloxacin and levofloxacin | 6 | 1 | 5 |
| Antibiotics use over 24 h after MT, n (%) | 105(40.7) | 49(46.2) | 56(36.8) |
| Piperacillin-tazobactam | 87 | 42 | 45 |
| Cephalosporin antibiotics | 13 | 4 | 9 |
| Moxifloxacin and levofloxacin | 4 | 3 | 1 |
| Clindamycin | 1 | 0 | 1 |

Values were measured the peak body temperature within 24 hours following MT.

Abbreviations: BT, body temperature; MT, mechanical thrombectomy.
