# Supplementary material for: Association between hyperpyrexia and poststroke outcomes in patients with recanalization after mechanical thrombectomy: a retrospective cohort study
Source: BMC Neurol. 2021 Sep 21;21:365. doi: 10.1186/s12883-021-02400-8 (PMC8454168; doi:10.1186/s12883-021-02400-8)
Supplement: Supplementary file 4 — Additional file 4: Supplementary Table S2. Relationships between diagnosis of infection/antibiotics use and clinical outcomes at different time points after MT. [file 12883_2021_2400_MOESM4_ESM.docx]

**Supplementary Table 2** Relationships between diagnosis of infection/antibiotics use and clinical outcomes at different time points after MT

| **Parameter** | **mRS 0-2** | **mRS 3-6** | ***P* value** |
| --- | --- | --- | --- |
| Infection/antibiotics use within 24 h after MT | 10(12.0) | 41(23.4) | 0.032 |
| Infection/antibiotics use over 24 h after MT | 27(32.5) | 78(44.6) | 0.066 |

Values were measured the peak body temperature within 24 hours following MT.

Abbreviations: MT, mechanical thrombectomy.
